# Advances in 3D-Printed Surface-Modified Ca-Si Bioceramic Structures and Their Potential for Bone Tumor Therapy

**DOI:** 10.3390/ma14143844

**Published:** 2021-07-09

**Authors:** Linh B. Truong, David Medina Cruz, Ebrahim Mostafavi, Catherine P. O’Connell, Thomas J. Webster

**Affiliations:** 1 Department of Chemical Engineering, Northeastern University, Boston, MA 02115, USA; truong.li@northeastern.edu (L.B.T.); davidmedinacrz@gmail.com (D.M.C.); cathy.p.oconnell@gmail.com (C.P.O.); websterthomas02@gmail.com (T.J.W.); 2 Stanford Cardiovascular Institute, Stanford University School of Medicine, Stanford, CA 94305, USA; 3 Department of Medicine, Stanford University School of Medicine, Stanford, CA 94305, USA

**Keywords:** bioceramics, biocompatibility, calcium silicate, nanomaterials, osteosarcoma, cancer therapy, photothermal therapy

## Abstract

Bioceramics such as calcium silicate (Ca-Si), have gained a lot of interest in the biomedical field due to their strength, osteogenesis capability, mechanical stability, and biocompatibility. As such, these materials are excellent candidates to promote bone and tissue regeneration along with treating bone cancer. Bioceramic scaffolds, functionalized with appropriate materials, can achieve desirable photothermal effects, opening up a bifunctional approach to osteosarcoma treatments—simultaneously killing cancerous cells while expediting healthy bone tissue regeneration. At the same time, they can also be used as vehicles and cargo structures to deliver anticancer drugs and molecules in a targeted manner to tumorous tissue. However, the traditional synthesis routes for these bioceramic scaffolds limit the macro-, micro-, and nanostructures necessary for maximal benefits for photothermal therapy and drug delivery. Therefore, a different approach to formulate bioceramic scaffolds has emerged in the form of 3D printing, which offers a sustainable, highly reproducible, and scalable method for the production of valuable biomedical materials. Here, calcium silicate (Ca-Si) is reviewed as a novel 3D printing base material, functionalized with highly photothermal materials for osteosarcoma therapy and drug delivery platforms. Consequently, this review aims to detail advances made towards functionalizing 3D-printed Ca-Si and similar bioceramic scaffold structures as well as their resulting applications for various aspects of tumor therapy, with a focus on the external surface and internal dispersion functionalization of the scaffolds.

## 1. Introduction

Primary bone cancers, also called sarcomas, have an annual worldwide occurrence of 3.4 million cases [1]. As such, osteosarcoma has become one of the most common primary malignancies in adolescence, along with leukemia and lymphoma [2]. According to the American Cancer Society, the overall 5-year survival rate of osteosarcoma of all stages is 60%. Metastatic osteosarcomas are associated with therapeutic resistance and low survival rates, a 19% 5-year survival rate, and 40% of patients suffering from a secondary metastasis [3]. The predominant treatment strategies against osteosarcoma all involve surgeries, provided that the tumor is localized and operable. Usually, surgeries for osteosarcoma patients can be divided into three groups: limb-sparing surgery, amputation, and rotationplasty. Limb-sparing surgery is the preferable operation, where only the cancerous tissues are removed, and full or partial function in the extremities is preserved [4]. However, if the tumor is aggressive or has begun to spread, amputations may be needed [5]. A rotationplasty offers both the removal of aggressive sarcoma and the preservation of normal function in the lower extremity by converting the ankle joint into the knee joint, followed by the attachment of a prosthetic [6]. Despite advances in surgical techniques and equipment, some tumors remain inoperable, and all surgical procedures carry risks of bleeding and infections. Another area of concern remains the long healing time and potential deformity caused by bone defects from surgeries, which adding tedious rehabilitation time and lowering the patient’s quality of life [7].

In addition to surgical strategies, neoadjuvant and adjuvant therapies are usually utilized, consisting mostly of chemotherapy with some radiation therapy considerations. Chemotherapy refers to the use of designed drugs to target a tumor cell’s differentiating mechanisms to inhibit tumor growth. Neoadjuvant chemotherapy, prescribed before surgery, serves multiple purposes: inhibiting tumor’s progression, evaluating the clinical response, and providing information for surgical planning [8]. In addition, adjuvant chemotherapy was shown to improve the survival rate after surgeries, but recent evidence questioned the efficacy of this approach. Notably, chemotherapy for osteosarcoma has not advanced much in the last decades, with little success in novel therapeutic agents outside of the four traditional drugs: methotrexate, doxorubicin, cisplatin, and ifosfamide [9]. This stagnation has prevented chemotherapy from drastically improving prognosis in osteosarcoma patients, not to mention the side effects associated with chemotherapeutic protocols. Meanwhile, radiation therapy uses high-energy rays to focus on the cancerous tissues to kill these tumor cells. This management method, usually performed concurrently with chemotherapy, is suitable for patients with inoperable tumors or those who receive incomplete removal of cancerous tissues. However, multiple types of research have questioned the benefits of radiation therapy, with little improvements in local tumor control and overall survival rates considering healthy tissue damage associated with such treatments [10,11]. Overall, while advances in osteosarcoma have shown remarkable improvements in prognosis, novel treatment methods should be investigated to improve the patient’s outcome and quality of life.

Among the new treatment methods, photothermal therapy (PTT) for localized treatment of bone tumors presents a promising approach. Using appropriate nanomaterials can efficiently raise temperature through energy delivered via light waves such as ultraviolet light or near-infrared (NIR). The treatment can induce apoptosis or necrosis in cancer cells by reactive oxygen species (ROS) pathways, cell membrane disruption, and immune system activation [12,13]. Therefore, PTT has emerged as an ideal method for localized tumor treatment, as damage to cancerous tissues can occur at 41 °C [14]. An apparent concern was the damage that could occur in surrounding healthy tissues, and thus the use of appropriate nanomaterials should be implemented to enhance the photothermal effect to targeted tumors. Various materials have been demonstrated to exhibit desirable light sensitivity properties for PTT, including metallic nanostructures, metallic oxides, sulfides, graphene derivatives, and organic nanostructures [15,16]. As a field, PTT provides the tools to treat osteosarcoma, especially regarding tumors whose locations are difficult to operate or target otherwise.

On the other hand, bioceramic (BC) scaffolds display good potential for osteosarcoma applications, especially with the goal of bone tissue regeneration. Then, BC is a class of inorganic biomaterials that can function as scaffolds for bone and tissue engineering due to their non-toxicity, inertness, strength, and stability. These materials include but are not limited to alumina, zirconia, glass ceramics, and calcium-based materials. Importantly, BC materials can produce scaffolds that are remarkably bioactive, resorbable, and osteoinductive, thus improving bone regeneration [17,18,19,20]. The osteogenesis effects of BC materials are the driving force behind its existing applications in dental settings and its rising research in bone tissue engineering. In osteosarcoma therapy, BC scaffolds were investigated for post-operative tissue healing, bone defects management, and bifunctional delivery with chemotherapeutic agents [21,22,23]. Specifically, Calcium-Silicate (Ca-Si) BC has been extensively studied in the literature, allowing a nearly perfect promotion of osteoblast differentiation and proliferation [24]. The calcium silicate BC can induce bone-like formation by releasing Ca^2+^ and Si^4+^ bioactive ions, enhancing its osteogenesis, promoting bone marrow stromal cell proliferation, and participating in bone mineralization [25]. Within this class of materials, the introduction of multiple formulations and modifications, such as those made of wollastonite (CaSiO_3_), diopside (CaMgSi_2_O_6_), and akermanite (Ca_2_MgSi_2_O_7_), were also studied [26,27].

From the promises of PTT in osteosarcoma therapy and the properties of Ca-Si BC materials in bone-tissue regeneration, bifunctional Ca-Si scaffolds with photothermal functionalization present novel and consequential directions for research. In terms of synthesis, traditional fabrication methods include gas foaming, fiber bonding, freeze-drying, phase separation/inversion, and particulate leaching. However, these approaches do not allow for the easy control of features in the final structure, such as pore shape, geometry, porosity, and interconnectivity [28]. Thus, 3D printing techniques become a feasible alternative, with significantly greater control of scaffold properties and a tunable design for different applications [29]. The final product is a scaffold that allows for the effective transport of nutrients, oxygen, waste, and growth factors while favoring the proper in-growth of bone tissue. Consequently, the ability to manipulate and incorporate materials into scaffolds makes 3D printing a promising method for BC production in the field [30,31,32].

Research has led to two main approaches to incorporate these materials onto Ca-Si BC scaffolds: external surface and internal dispersion functionalization. This review will discuss the current advances in each strategy in synthesizing 3D printed bio-ceramic scaffolds composed of Ca-Si materials for photothermal osteosarcoma therapy, summarizing some of the most critical surface modifications made to Ca-Si BCs.

## 2. External Surface Functionalization of Scaffolds

**Figure 1:**
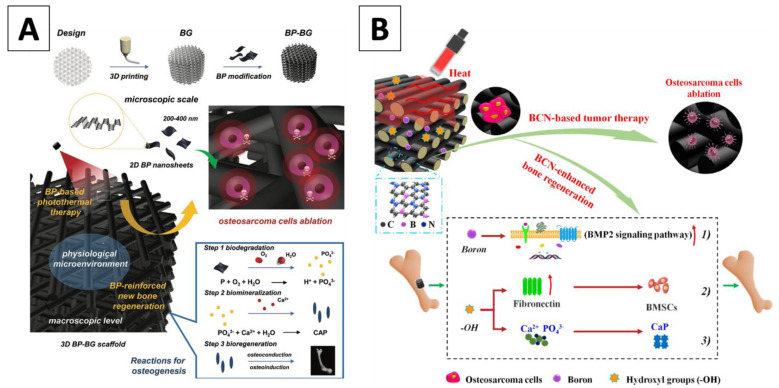
(**A**) Illustration of the fabrication process for BP-BG scaffold, highlighting the therapeutic strategy around the use of the scaffold for the successful removal of osteosarcoma, which was followed by osteogenesis through three steps: biodegradation, biomineralization and bioregeneration (Yang 2018) (**B**) Schematic showing the integrated strategy of BCN-AKT scaffolds for triggering photothermal therapy and repair of tumor-initiated bone defects (Zhaoa 2020). Abbreviations: BP nanosheets combined with 3D printed bioglass (BP-BG); 2D borocarbonitrides nanosheets combined with akermanite in a 3D bioglass (BCN-AKT).

External surface functionalization involves coating of highly photothermal materials onto 3D-printed BC structures. This strategy proposes the functionalization of a scaffold after 3D printing, hence enhancing the overall photothermal properties of the platform. Ideally, the perfect agent will be a nanomaterial with high biocompatibility and osteogenic activity which can satisfy a prominent photothermal effect [33]. For instance, a mussel-inspired scaffold with self-assembled nanostructures was prepared to provide an efficient tumor therapy while promoting bone regeneration. The authors fabricated a 3D scaffold structure of Nagel (Ca_7_Si_2_P_2_O_16_) coated via self-assembly with a uniform Ca-P/polydopamine nanolayer, intending to provide a boost to the already well-reported bone-forming activity of the substrate BC (Figure 1A). The authors proposed an assembly mechanism of the nanolayers in three steps: the dissolution of Ca and P ions in solution, nucleation site formations due to negatively-charged groups, and simultaneous Ca-P mineralization and dopamine polymerization, forming the final observed structure (DOPA-BC). After characterization, the photothermal properties of DOPA-BC scaffolds were quantified by the degree of temperature change under NIR irradiation. During in vitro testing using NIR radiation, the scaffold was reported to exhibit excellent conversion from NIR energy to heat, reaching up to 50 °C in wet conditions (Figure 1B). This excellent performance was proposed in previous studies, where the self-polymerization process correlated with high photothermal conversion efficiency [34]. Significantly, compared to non-modified BC, DOPA-BC showed the ability to reach a higher temperature under the same conditions, favorable for photothermal therapy. Then, an in vivo study was conducted to observe the photothermal effect by covering the scaffolds with different pork thicknesses. The experiment showed desirable and effective photothermal performance for tissues with up to 7.5 mm in thickness, providing a tool to impact tumors with difficult locations. The authors applied DOPA-BC scaffolds into two different tumor cells models, Saos2 (osteosarcoma) and MDA-MB-231 (breast cancer), and they observed that the cell mortality after NIR treatment reached ∼88.2% and ∼80.4%, respectively. In order to validate these findings, in vivo studies with tumor Balb/c nude mice and large bone defects models in rabbits were conducted. At the end of 14 days, the tumor size in the control group grew up to six times in volume compared to day 0, while the DOPA-BC-NIR treatment showed a decrease in size after day 4. Evidently, the hyperthermia effect from the NIR irradiation onto the DOPA-BC scaffold significantly inhibited tumor cell proliferation and induced apoptosis, thus revealing a mechanism of therapeutic action [35]. As for the osteogenesis properties of DOPA-BC scaffolds, the authors revealed that rBMSCs cells proliferated well in both pure BC and DOPA-BC structures. Specifically, femoral defects in rabbits were treated either with BC or DOPA-BC, and a short NIR irradiation exposure was performed to evaluate the potential for long-term tissue regeneration. The results indicated two takeaways: the DOPA modification enhanced bone regeneration in rabbits compared to both unmodified BC and untreated defects and the NIR irradiation did not show any hindrance on the overall osteogenesis. Eventually, it was established that the Ca-P/polydopamine nanolayers played an important role in initiating the response of bone cells and bone tissues in vivo. The authors concluded that this behavior was associated with: (a) an improvement of surface roughness, hydrophilicity, and bioactive functional groups from a Ca-P/polydopamine nanolayer that can support the proliferation of cells [36]; and (b) the catechol groups in polydopamine are associated with a significant improvement of apatite nucleation and mineralization on the nanostructured surface [37]. Therefore, the prepared scaffold with mussel-inspired nanostructures can be used to treat tumor-related bone defects in combination with NIR application while promoting the regeneration of bone tissues after surgical removal of tumor tissue and decreasing the risk of tumor recurrence through localized photothermal therapy [38].

Similarly, black phosphorus (BP) was successfully integrated onto a 3D-printed bioglass (BG) of the Ca-Si-P composition framework. The BP-BG scaffolds were assembled by surface modification of BP onto 3D-printed BG scaffolds, with the final thickness of the BP layer less than 10 nm. The 3D-printing technology enabled specified macropores and desired, disordered surface characteristics of the scaffolds, which may assist in bone tissue regeneration by providing a desirable attachment surface between the cells and the assembly [39,40]. Elemental analysis showed that phosphorus was concentrated on the strut surface of the scaffolds, which favored the regulation of the photothermal performance and osteogenic capability. Under NIR treatment, the change in temperature of BP-BG structure was effective and fast, reaching 68.7 °C within 5 min. During the increase in temperature, the photothermal efficiency of the BP-BG scaffold remained robust, thus suggesting a sustained therapeutic potential for hyperthermia treatment. The authors applied these designs onto cancerous Saos-2 cells in vitro. The adherence of Saos-2 cells onto the scaffolds was observed, highlighting the cytocompatibility of BP-BG structure and serving as a preview for its promise in bone tissues reintegration. When NIR irradiation was applied, the viability of Saos-2 cells decreased significantly, with less than 20% of cells remaining at a power density of 0.8 W/cm^2^. Notably, in the following animal study with a nude mice tumor model, photothermal therapy was performed at 1 W/cm^2^ and several promising pieces of evidence were obtained. First, the BP-BG scaffold was able to reach a higher temperature compared to the unmodified BG scaffold within 300 s of treatment. Specifically, the BP-BG scaffold was able to achieve a 55 °C temperature after 1 min, which was a rapid difference from the untreated BG structure, which did not achieve 40 °C in the same duration. Second, in the BP-BG-NIR group, the tumors were eradicated without recurrence within the observation period of 14 days, whereas BG-NIR and untreated tumors increased in size. Despite these promising results, the authors were concerned about the compositional changes and degradation of BP, but optimistic that the bio-oxidation of BP nanosheets can overcome this challenge via an in situ biomineralization process [41]. Therefore, the in situ biomineralization process of BP nanosheets was evaluated in vitro by dispersing them into simulated body fluid. Intriguingly, the authors found that large numbers of nanoparticles generated by this time-dependent biomineralized reaction can adhere onto the surface of the few remaining BP nanosheets, which may prevent these slight amounts of BP components from further corrosion [42]. Additional stoichiometric experimental results based on the EELS and EDS analyses provided evidence of a clear phosphorus-driven, calcium-extracted biomineralization process of the BP nanosheets, which greatly favored subsequent bone generation. Due to this biomineralization, subsequent studies to evaluate its osteogenesis and osseointegration provided supporting results. Qualitatively, a dense layer of hBMSCs was observed to adhere and proliferate on the scaffold, with characteristics consistent with healthy cytoskeletal tissues. Quantitatively, the usage of BP-BG scaffolds after 5 days of incubation resulted in an increase in bone volume and bone density, two robust indicators of osteoinduction. To validate these findings in an animal model, cranial defects in mice were treated with a BP-BG scaffold as well as a BG scaffold. A thin newborn osseous tissue was observed by week 8, and the scaffolds were eventually degraded. The authors hypothesized that the osteogenesis process was achieved through the migration of osteocytes from the surface to the center of the scaffold’s structure, simultaneously occurring with the degradation of the scaffold and subsequent closure of the defect. All in all, the authors concluded a 3.7-fold enhancement in osteoblast function on the BP-BG scaffolds compared to regular BG, thus arguing the vital importance of the BP nanosheets modification in assisting the proliferation of bone cells (Figure 1A) [43].

The ability of β-tricalcium phosphate (β-TCP) to promote desirable osteogenesis performance was well-established due to its robust bioactivity. Combined with the angiogenic and photothermal properties of Copper-tetrakis (4-carboxyphenyl porphyrin) (Cu-TCPP), an assembly was synthesized by the surface incorporation of Cu-TCPP onto 3D-printed β-TCP scaffold (Cu-TCPP-TCP). The rationality of the design included the photothermal Cu-TCPP sheet which can hinder the tumor’s progression, while bioactive β-TCP can repair the bone defects. Therefore, the 3D-printed β-TCP scaffold was generated, and the Cu-TCPP sheet was added onto the surface through a solvothermal process, with a final thickness of approximately 50 nm. The photothermal performance of the resulting assembly was evaluated under NIR irradiation in both wet and dry conditions. Previous studies indicated a 36.8% photothermal efficiency of Cu-TCPP, with was credited to the copper vacancies and the ultra-thin structure. Accordingly, the Cu-TCPP-TCP also exhibited fast and effective photothermal conversion, reaching 55 °C in wet conditions at a 0.9 W/cm^2^ power density. The authors also showed correlations between different variables to the maximum reachable temperature, including the concentration of TCPP and the power density. While TCPP-TCP did not inhibit LM8 osteosarcoma cells, the Cu-TCPP-TCP structure showed good anti-tumor properties in the presence of NIR treatment. Specifically, coupled with a 1.0 W/cm^2^ power density, the Cu-TCPP-TCP scaffold facilitated the killing of LM8, leading to a 10% tumor cell viability after 10 min. The tumor-killing performance was also correlated with different variables, as longer NIR radiation and higher power density resulted in lower cancer cell viability. The authors indicated that high temperature, documented to be higher than 48 °C, from the photothermal conversion damaged tumor cells by irreversible DNA fracture and protein denaturation. The Cu-TCPP-TCP structure reached 58 °C, thus it was effective in killing LM8 osteosarcoma cells. Subsequently, a subcutaneous bone tumor model was constructed in naked mice and Cu-TCPP-TCP scaffolds implanted into bone tumors on the backs of mice. Results showed that the bone tumors of the Cu-TCPP-TCP scaffolds in the presence of the NIR treatment group did not show continuous growth. Additionally, morphological changes in HBMSCs and HUVECs were studied, and their results showed that the extract solutions of the scaffold promoted the expressions of osteogenesis differentiation-related genes ALP, BMP2, OCN, and RUNX2. Finally, critical-sized femoral defects in rabbit models were constructed in order to evaluate the osteogenic performance of the structures. Results revealed that the Cu-TCPP-TCP scaffolds implanted into rabbit femoral defects promoted the regeneration of new bone tissues after eight weeks. Additionally, the authors argued that the Cu-TCPP-TCP scaffolds released bioactive ions, such as Cu^2+^ and Ca^2+^ in vivo, whose presence promoted the expressions of proteins critical toward the osteogenesis and osseointegration processes in bone tissues [44]. The authors then concluded that combining a metal-organic framework (MOF) material with porous BCs scaffolds offerred a new horizon and inspiration to fabricate multi-functional biomaterials [45].

Carbon-based materials, especially graphene and its derivatives, have also been used increasingly as coatings on 3D printed BCs scaffolds due to their osteoinductive, antibacterial, and mechanical strengthening properties [46]. Within osteosarcoma applications, these materials are also promising due to the robust NIR absorbance and subsequent robust photothermal conversion efficiency. In addition to this excellent photothermal profile, graphene and its derivatives also exhibit acceptable cytocompatibility and safety in living systems [47,48]. To study the potential combination of GO with BCs, Ma et al. conducted a study using β-TCP 3D-printed scaffolds, whose surface was coated graphene oxide (GO). As mentioned, 3D printing enabled the synthesis of bioceramic scaffolds with well-controlled porous characteristics. Subsequently, GO modification was performed on all pore-wall surfaces of β-TCP scaffolds, with GO wrinkles observed on the pore walls of the final structure, referred to as GO-TCP scaffolds. At an NIR radiation of 808 nm, the GO-TCP structure showed excellent photothermal absorbance, both under dry and wet conditions. The authors also provided correlations between this photothermal effect with GO concentrations, exposure duration, power density and scaffold size. During in vitro testing against MG-63 osteosarcoma models, the GO-TCP structure reduced the viability of cancer cells by 80%, while the non-modified TCP scaffold did not show any inhibition to MG-63 growth after 10 min of NIR treatment. The authors found that the photothermal temperature played a key role in killing tumor cells, consistent with other established studies. Subsequently, an in vivo tumor-bearing mice model was conducted and showed that tumor volume in the GO-TCP scaffolds group with irradiation significantly shrunk after photothermal treatment. The results showed that the tumor cell necrosis rate in GO-TCP scaffolds with the irradiation group reached up to 83.28% through quantitative analysis. To confirm the osteogenesis properties of the GO-TCP scaffold, the morphology and attachment of rBMSCs cells were studied, and cell attachment with a well spread morphology on the pore walls was observed. Similar results were noted during rabbit models with critical-sized calvarial defects. After 8 weeks, histological analysis showed the presence of new bone tissues formed both in the periphery and the center of the defects, attributed to the infiltration of cells via the macropores. In addition, short-term NIR irradiation showed no disadvantageous effect on the long-term proliferation and migration of bone marrow mesenchymal stem cells. Therefore, GO modification showed potential for both anti-tumor therapy in bone cancers using the photothermal treatment and the eventual tissue regeneration afterward [49].

Another approach to functionalize 3D bioceramic scaffolds involves using borocarbonitrides (BCN) nanomaterials due to their high surface area, electrocatalytic activity as well as electrical and thermal conductivity [50]. Furthermore, BCN materials contain carbon and graphene derivatives and therefore can deliver exceptional photothermal efficiency. Coupled with the inherent process of mineralization and osteogenesis where boron acts as a crucial element, the BCN functionalization of BC scaffold presented an excellent approach for both photothermal tumor therapy and bone regeneration in osteosarcoma application [51]. Inspired by these possibilities, Zhao et al. used BCN nanosheets integrated into 3D-printed Akermanite (AKT) (composition: Ca_2_MgSi_2_O_7_), which were studied as a desirable BC material for bone tissue regeneration due to their osteogenic activity and biodegradability [52]. The authors used a boric acid solution that was continuously evaporated for crystallization during synthesis, so the boric acid crystals formed sheet-like structures on the AKT surface via hydrogen bonds. Boron oxide was then generated through boric acid decomposition, followed by the carbonization process, with the final ultra-thin sheet with a lateral size of around 20 μm. Under an 808 nm NIR treatment, BCN nanosheets showed enhanced, power-dependent photothermal properties, reaching an exceptionally high temperature of 156 °C after 5 min. To confirm, in vitro tests were performed with osteosarcoma (MNNG/HOS) cells, which an ultra-low viability of 11% was observed, in contrast with a near 100% viability in other groups. For in vivo experiments, tumor-bearing male BALB/c nude mice were selected as models, with BCN modified scaffolds treated with 10 min of NIR irradiation, along with other comparative groups. As a result, the BCN scaffolds facilitated a rapid rise in the tumor temperature, reaching 52 °C. The tumors in this group were eliminated completely, while other groups exhibited rapid growth in volume. The authors continued to study the biomineralization effect of the BCN-AKT scaffold, and they observed deposition of nanoparticles of calcium and phosphorus after 3 days of immersion. Such observations supported the hypothesis of in situ mineralization to form Ca-P nanoclusters and confirmed the capability of BCN for biomineralization. Osteoproliferation assays were then performed with BMSCs cells throughout a 7 days culture period, with data showing greater proliferation on the BCN-treated scaffolds than that of the AKT scaffolds. The results revealed that the surfaces stimulated a rapid attachment and growth of the surrounding cells, a desirable hallmark for long-term bone regeneration. This advantage was further validated in femoral defects models in rabbits. Compared with the control group, new bone tissues in the BCN scaffolds group infiltrated the internal pore structures, and the biodegraded scaffold sites displayed prominent osteogenic activity. Therefore, after all the presented results, the authors concluded that the superiority of their design was related to four main key factors: (a) their scaffolds showed a significantly higher adsorption of BSA than AKT scaffolds due to increased surface area from nanosheets, thus modulating cell behaviors [53]; (b) the expression of fibronectin (FN) on the scaffolds was significantly upregulated, which is known to enhance cell attachment and promote osteogenesis [54]; (c) the biomineralization capability of BCN nanosheets was higher, driving osteogenic differentiation; and (d) the release profiles demonstrated that B element was successfully introduced into the BCN nanosheets, hence promoting mineralization and osteogenic differentiation via the activation of the BMP2 signaling pathway [55]. Consequently, the authors successfully designed a promising therapeutic approach for osteosarcoma treatment by loading BCN nanosheets onto the surface of 3D-printed AKT scaffolds (Figure 1B) [56].

## 3. Internal Dispersion Functionalization of Scaffolds

**Figure 2:**
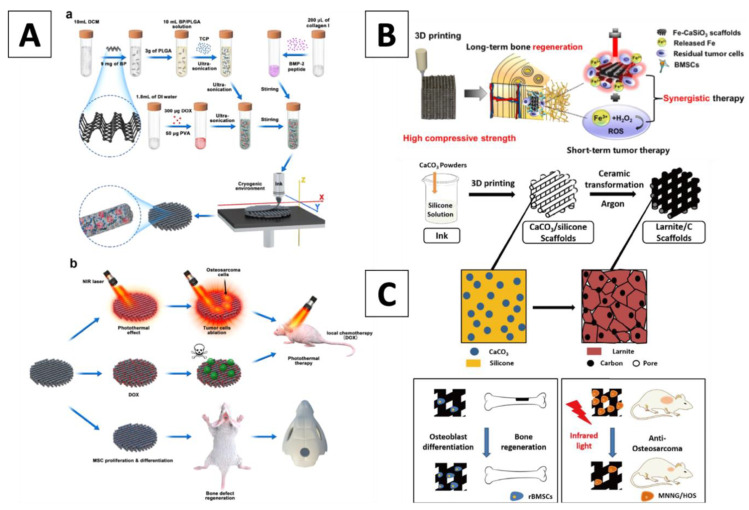
(**A**) Illustration of the cryogenic 3D printing process for the generation of multi-functional scaffolds along with their biomedical application; (a) Synthesis process and 3D printing of multi-functional scaffolds; (b) Process of tumor tissue ablation in mice by mean of photothermal therapy and localized chemotherapy, as well as total regeneration of cranial bone defects (Wang 2020); (**B**) Illustration of the fabrication of Fe-CaSiO_3_ scaffolds and their biomedical application for long-term bone regeneration by offering a synergistic therapy with short-term tumor therapy (Ma 2018); (**C**) Illustration for the fabrication process of the larnite/C scaffolds and their biomedical applications (Fu 2020).

Aside from functionalizing a 3D-printed BC scaffold’s surface, another strategy to deliver photothermal properties into the structure is to embed photothermal agents into the BC solution before printing the composite. This approach ensures the uniform distribution of photothermal properties and limits the degradation of the coating material. These photothermal agents include metals and nonmetal agents, as presented in Table 1 and Figure 2, as well as in the several examples discussed below.

For example, Ma and colleagues designed a composite consisting of Fe-doped wollastonite (CaSiO_3_) which was built using a 3D printing method. The iron nanoparticles (FeNPs) acted as a photothermal agent due to its localized surface plasmon resonance. Additionally, the authors were looking for an impact of the release of iron ions from the structure and their ability to act as catalysis agents in H_2_O_2_ decomposition inside the targeted tumors, leading to the production of reactive oxygen species (ROS) [57]. Mechanical tests on the Fe-CaSiO_3_ composite scaffolds demonstrated that they could withstand the pressure from in vivo orthopedic procedures, while exerting photothermal activity under irradiation by an 808 nm NIR laser due to the localized surface plasmon resonance properties of the structure [58]. For cell studies, Saos2 osteosarcoma and rBMSCs cells were exposed to the composite and the production of ROS was observed, which was linked to the release of Fe ions. Furthermore, irradiation rates of 15 min and subsequent incubation of four hours with the tumoral cells showed a mortality rate of 91.4%, with a cell death mechanism dependent on the temperature, which led to cell membrane collapse and protein denaturation, among other cell dysfunctions [49]. Furthermore, significant and rapid lipid oxidation as well as protein and DNA damage were observed and linked to the overproduction of ROS [59]. Subsequently, the scaffolds were implanted in the center of tumors in mice and exposed to the conditions employed in vitro. Tumor cells presented fibrosis and nuclei dissolution, which was linked to the combined photothermal and ROS mechanisms triggered by the composites. Therefore, the authors identified that their scaffolds could be utilized to selectively kill tumor cells while causing minimal damage to normal cells and reported two key factors for such selectivity: (1) the production of ROS, which did not harm healthy cells to the same extent as cancer cells, mainly because of the higher amounts of H_2_O_2_ in tumor cells [60]; and (2) the fact that certain tumor cells, such as breast cancer cells, displayed more transferrin receptors than healthy cells. The presence of these membrane glycoproteins, whose function is to mediate cellular uptake of iron, clearly lead to higher intracellular concentrations of iron ions in the tumor cells [61]. Finally, the scaffolds were evaluated for their effect on the proliferation of rBMSCs over five days, suggesting good biocompatibility of this material. Genetic studies targeting the expression of typical markers of osteogenic differentiation showed a dramatic upregulation on day seven, attributed to the presence of CaSiO_3_, as it can enhance the cross-talk between endothelial cells and bone marrow stromal cells and further stimulate vascularization and osteogenic differentiation [62]. The in vivo osteogenic capability was tested on rabbits that were exposed to irradiation at a power density of 0.8 W/cm^2^ for 10 min, with the aim to verify that the short-term photothermal therapy had no adverse effects on long-term bone regeneration. After 8 weeks, desirable bone compatibility and conductivity were observed and were linked to the surface reaction of CaSiO_3_ with the surrounding fluids and the formation of bone-like apatite on the surface of the scaffolds [63]. The authors found that the ceramic phase exhibited enhanced structural degradation, while the release of Si ions was associated with collagen synthesis and bone mineralization, as well as osteoblast proliferation and differentiation [64]. Therefore, the prepared Fe-CaSiO_3_ scaffolds were presented as both versatile and efficient biomaterials for the treatment of bone cancer [65]. Along the same line of research, a different group used Fe doping on AKT powders. The Fe-AKT scaffolds, which presented a tailored porous structure, were fabricated by a 3D printing strategy. An initial in vitro weight loss test suggested that Fe-doped AKT scaffolds possessed good degradability rate and that the bioactive ions dissolved from AKT, mainly Mg and Si ions, played vital roles in osteoblastic differentiation [66]. Therefore, the authors found that their BC might have good bioactivity due to the dissolved Fe ions, which can stimulate the proliferation and differentiation of bone marrow-derived stem cells [67]. After characterization, an alternating magnetic field was applied, triggering a sudden increase in temperature. Simultaneously, the photothermal performance was investigated by exposing the surfaces to irradiation from an 808 nm laser beam. Therefore, the authors demonstrated the presence of both photothermal and magnetothermal effects on the scaffolds, which would prevent the need for a higher dose of irradiation, reducing harmful effects on healthy tissues. To test such effects, murine osteosarcoma LM-8 cells were cultured on the scaffolds, whose temperature reached 43 and 47 °C after either magnetothermal or photothermal exposures, respectively. On the other hand, the temperature of the scaffold after photothermal/magnetothermal dual-mode treatment reached 53 °C. Cell viability after photothermal exposure was 59.2%, compared to 81.6% after the magnetothermal treatment. Surprisingly, cell viability after the dual photo/magnetothermal treatment was only 2%. Afterward, rBMSCs cells were cultured on different scaffolds for 1, 3, and 5 days. Results showed that cells cultured on Fe-AKT scaffolds presented a significantly higher proliferation rate than those on control scaffolds made of just AKT, hence revealing an upregulation of osteogenic differentiation of bone mesenchymal stem cells. This event was attributed to the presence of Fe ions, which can upregulate the expression of bone-related genes at certain concentrations [67]. Therefore, due to its superior hyperthermal synergy, the Fe-AKT scaffolds triggered an improved antitumor efficiency compared to a single-mode of treatment in vitro [68].

Expanding further on the metal-based doping of 3D printed BC scaffolds, Liu et al. developed a bioactive glass-ceramic powder that was doped with the metals Cu, Fe, Mn and Co, and then was 3D-printed in the hydrophilic non-ionic surfactant Pluronic F-127. Excellent photothermal performance was observed and attributed to the defects in the transition metal oxides structures [69], which induced surface plasmon resonances. Therefore, the authors controlled the plasmon resonance by changing the free carrier concentration in the prepared structures [70]. For the experiments, an 808 nm laser irradiation was applied for 15 min to the scaffolds combined with Saos-2 cells, showing survival rates of 1.2%, 17.6%, 37.4% and 43.1% for the Cu-, Fe-, Mn- and Co-based scaffolds, respectively, as compared to 98.1% in the control group. Interestingly, the authors found that an increase in laser power density led to a decreased survival rate of Saos-2 cells, determining that the power density of 0.54 W/cm^2^ was enough to cause cell apoptosis. After several tests, it was concluded that the antitumor effect of scaffolds, by means of photothermal exposure, exhibited the following trend based on the doping elements: Cu- > Fe- > Mn > Co. In vivo studies were performed in mice, revealing that the Cu-based scaffolds possessed the most remarkable tumor therapy effect. Lastly, the scaffolds were co-cultured with rBMSCs cells and results showed that all of the five types of scaffolds promoted the osteogenic differentiation of rBMSCs, with the ionic dissolution of Ca and Si allowed for better differentiation of osteoblasts. Gene regulation studies showed that Cu^2+^ and Co^2+^ ions were able to simulate a hypoxic environment by stabilizing the hypoxia-inducible factor 1-alpha, also known as HIF-1-alpha [71,72] Furthermore, Mn release, as an important component of bone and cartilage matrix, was able to improve the affinity of integrins and ligands [73], while Fe played an important role in the maturation of collagen [74]. After a careful analysis of the data, the authors identified the main advantages of their scaffolds: (1) effective control of the photothermal activity can be easily achieved by altering the doping element categories, contents, and the laser power densities; (2) the element-doped scaffolds could function as locally targeted photothermal agents; and (3) the scaffolds can be implanted and function as locally targeted photothermal agents [75].

Other than metal elements as doping agents, nonmetal elements can also be used to dope the BC composition before printing. For instance, Wang et al. employed hydrophobic BP nanosheets, doxorubicin hydrochloride (DOX), and the hydrophilic bone morphogenetic protein-2-like osteogenic peptide (also known as P24) to build a novel scaffold with photothermal properties. These components were incorporated into water-in-oil composite emulsions to form printing inks that were used as raw materials for the layer-by-layer construction of multi-functional scaffolds through micro extrusion-based 3D printing in a cryogenic environment. BP nanosheets were built as the perfect photothermal agent, hence when irradiated, the temperature of the scaffolds increased from room temperature to 60 °C within 10 min. Results revealed the in vitro release behavior of DOX from the scaffolds, which had an accelerated release profile when NIR irradiation was applied. Additionally, when NIR was applied for 5 min, nearly all MG63 cells on the scaffolds were killed after one-day of culture. Besides, upon NIR irradiation, a clear tumor ablation was found in mice models, showing a significant reduction in tumor volume within the fourth day. A significantly low tumor recurrence rate was also demonstrated, suggesting the scaffolds had efficient photothermal and chemotherapy properties that worked simultaneously in a synergistic way. In addition, a more sustained DOX release at the bone regeneration stage was found and was attributed to the presence of BP nanosheets, hence becoming favorable for the in vitro growth and osteogenic differentiation of rBMSCs cells [76]. Consequently, the authors confirmed that the superiority of their scaffolds was associated with: (1) a lowered DOX release due to the non-specific/physical adsorption of DOX onto BP nanosheets; and (2) a more sustained peptide release, supporting enhanced bone formation [77].

Another nonmetal agent used as a doping agent in bulk BC is carbon, offering important improvements that were explored by Fu and colleagues, who developed a novel scaffold with C as a doping agent. Briefly, the authors used polysilsesquioxane silicone, an organic-inorganic hybrid material with excellent heat and weather resistance, and CaCO_3_ active filler powders, which were homogeneously mixed for 3D printing. Then, a ceramic transformation was completed under an argon atmosphere to form a free carbon-embedding larnite scaffold. Larnite was used as the bioactive ceramic due to its excellent apatite formation ability and promotion to osteogenic gene expression [78]. On the other side, the employment of free carbon exhibited good biocompatibility while triggering low toxicity for cells [79]. The presence of C allowed also for the absorption of NIR light, hence promoting a high photothermal conversion and leading to an efficient cell death mechanism in tumors. An in vitro and in vivo photothermal performance study was completed, and the results showed that the surface temperature on the larnite scaffolds increased to around 63 °C. The viability of MNNG/HOS osteosarcoma cells was significantly decreased with the increase in NIR light power intensity. A subsequent in vivo study reported that after a 14-day photothermal treatment, the tumor volume can significantly decrease, while promoting high osteogenic capability. The authors discussed that the presence of C in the larnite scaffold further enhanced new bone formation by upregulating the expression of osteogenic genes (such as Runx-2, ALP, OCN, and BSP) as well as extracellular matrix mineralization [80]. Therefore, new bone formation can be dramatically improved by offering a synergy between free carbons and larnite in the form of novel scaffolds [81].

## 4. Challenges and Prospects

While multiple types of research have shed light on the promise of combining 3D-printed BC scaffolds with functionalizing materials to enhance the effectiveness of osteosarcoma treatment, the road to clinical translation is still challenging [82]. Some of these obstacles come from the ceramic materials’ inherent undesirable properties, such as their brittleness [83]. Without ideal physical properties, concerns about the BC material biocompatibility and durability in clinical settings persist. A particular challenge to overcome remains the longitudinal understanding of the scaffold in the body, especially considering the release of bioactive ions. While desirable, a high concentration of bioactive ions can result in osteolysis and inflammation [84]. Furthermore, functionalized BC scaffolds need special attention due to the eventual release of doping materials, and the safety profile of all components—BC scaffold, doping molecules and assembled structure—need to be understood. Therefore, careful considerations to material composition, concentration and designs are needed in order to utilize BC scaffold as a means to limit the tumor progression and expedite osteogenesis.

With respect to 3D printing as a synthesis method, most challenges involve designing and manufacturing ceramic scaffolds, such as the complexity of the design process, the high need for customization, in vivo bio-incompatibility and printing resolution [85]. Limitations involving the printers’ capabilities, such as resolution, material viscosity and speed, remain as bottlenecks for both the quality and scalability of functionalized BC scaffolds [86]. A more efficient and safer 3D printing process would be highly desirable to push the field toward clinical applicability.

To address these concerns and to advance the future of 3D printed BC scaffolds in the management of osteosarcoma, a progressions in materials, manufacturing techniques and clinical validations are necessary. The continuous exploration of new BC scaffolds formulations is ongoing with the goal of promoting stability, biomimicry and osteogenesis [87,88]. At the same time, the development of novel doping molecules to functionalize such scaffolds presents a robust field of research, with the objective to optimize the photothermal performance of the overall structure for efficient tumor removal [89,90]. Combining these materials’ advances with expected improvements in 3D printing technologies such as stereolithography, selective laser sintering and 4D printing [91,92,93,94], the quality and scalability of functionalized BC scaffolds could be ready for clinical translation.

Yet, only a limited number of rigorous clinical studies, both in larger animal models and early human trials, were performed to validate the risks and benefits of 3D printed functionalized BC scaffold photothermal treatments for cancer indications. Currently, 3D-printed structures alone (without photothermal properties) have made their way through the clinical translation, with most studies addressing the reconstruction of bone fractures. Specifically, detailed images of a patient’s tissues have been captured and a 3D design of an implant is constructed accordingly to be ready for immediate implantation via surgery [95]. Within cancer applications, recent advances include the use of 3D-printed template-assisted CT-guided radioactive seed for radiation therapy, where the printed template was personalized and supported more targeted radiation. However, these clinical studies utilizing 3D-printed platforms are still in their early stages, with a small number of patients, and might not be indicative of their long-term and wide-spread clinical performances [96,97]. In bone regeneration applications, large animal models had been studied with a 3D-printed bioceramic scaffold, implanted via surgical procedures. Promisingly, these scaffolds performed well in animals as large as equine models, showing both biodegradability and osteogenesis [98]. Similar observations were seen in the clinics, where 3D-printed bioceramic implants were seen as a potential state-of-the-art approach for treating bone defects and bone fusions [99,100]. However, presently, such modified 3D-printed bioceramics have not advanced into human trials. As a whole, photothermal therapy is still in its early stage compared to established chemotherapeutic treatments, and until the clinical validation of photothermal therapy has advanced, additional features are unlikely to be considered due to a lack of regulatory structure and evidence [101]. With modification toward bioceramic structures, a rigorous evaluation in its regulatory pathways is needed, as the resulting treatment may be seen as a device-drug combination product, adding another layer of complexity to clinical translation. Such high development costs, uncertain clinical performances, and questionable manufacturing scalability have somewhat discouraged the translation and commercialization in this particular field. However, there is hope that with more safety data, efficacious designs and 3D-printing advancements, these challenges can be overcome in the near future.

## 5. Conclusion

3D printing has become a popular manufacturing process to produce BC scaffolds due to its versatile customization concerning shape, pore size, geometry, and interconnectivity. Functionalization gives Ca-Si BC scaffolds desirable photothermal properties, in addition to their existing tissue regeneration properties. Both external and internal functionalization of 3D-printed BC composites can provide for more effective photothermal efficiency, leading to improved antitumor properties as seen in both in vitro and in vivo settings. Despite the observed benefits, 3D printed and functionalized BC scaffolds face several challenges regarding their inherent properties, manufacturing capacity and a long road toward clinical viability as an approved treatment for osteosarcoma. However, the advantages and promises of these ceramic assembles must be acknowledged and explored, as they could be the solution that society badly needs for alleviating the pain of osteosarcoma patients.

## Figures and Tables

**Table 1:** Summary of current BC 3D scaffolds with their functionalizing agents.

Functionalization	BC Composition	Functionalizing Agents	The Temperature Achieved during NIR Radiation	In-Vitro Antitumor (% Cancer Cell Viability) during NIR (W/cm^2^)	Ref.
External Surface	Nagel (Ca_7_Si_2_P_2_O_16_)	Ca-P/polydopamine	50 °C at 0.34 W/cm^2^	0.8–1%/0.38	(Ma and Luo, et al, 2016)
	Bioglass (Ca-Si-P)	Black Phosphorus	70 °C at 1 W/cm^2^	<5%/1	(Yang 2018)
	Beta-tricalcium phosphate	Copper-tetrakis (4-carboxyphenyl) porphyrin (Cu-TCPP)	55 °C at 0.9 W/cm^2^	<10%/1	(Dang 2020)
	Beta-tricalcium phosphate	Graphene Oxide	40–90 °C at 0.36 W/cm^2^	14%/0.36	(Ma and Jiang, et al, 2016)
	Akermanite (Ca_2_MgSi_2_O_7_)	Borocarbonitrides	55 °C at 0.35 W/cm^2^	11%/0.3	(Zhaoa 2020)
Internal Dispersion	Wollastonite (CaSiO_3_)	Iron (Fe)	50 °C at 0.6 W/cm^2^	8.6%/0.6	(Wu 2006)
	Akermanite (Ca_2_MgSi_2_O_7_)	Iron (Fe)	45 °C at 0.7 W/cm^2^	40%/0.7; 2%/0.7 in combination with 896.8 A/m magnetic field	(Zhang 2017)
	Glass ceramic (Ca_0.25-0X_P_0.05_Si_0.25_)	Copper, Iron, Cobalt, Manganese	40–55 °C at 0.36 W/cm^2^	0–10%/0.54	(Qiu 2018)
	Beta-tricalcium phosphate	Black phosphorus—doxorubicin hydrochloride	50 °C at 1.5 W/cm^2^	No in vitro data, but tumor volume decrease is recorded In vivo	(Gou 2005)
	Larnite (Ca_2_SiO_4_)	Free Carbon from CaCO_3_	50 °C at 0.75 W/cm^2^	50%/0.75	(Mehranfar 2019)

## Data Availability

Not applicable.
